# A ruthenium-based 5-fluorouracil complex with enhanced cytotoxicity and apoptosis induction action in HCT116 cells

**DOI:** 10.1038/s41598-017-18639-6

**Published:** 2018-01-10

**Authors:** Valdenizia Rodrigues Silva, Rodrigo S. Corrêa, Luciano de Souza Santos, Milena Botelho Pereira Soares, Alzir Azevedo Batista, Daniel Pereira Bezerra

**Affiliations:** 1 Gonçalo Moniz Institute, Oswaldo Cruz Foundation (IGM-FIOCRUZ/BA), Salvador, Bahia 40296-710 Brazil; 2 Department of Chemistry, Federal University of Ouro Preto, Ouro Preto, Minas Gerais 35400-000 Brazil; 3 Center of Biotechnology and Cell therapy, Hospital São Rafael, Salvador, Bahia 41253-190 Brazil; 4 Department of Chemistry, Federal University of São Carlos, São Carlos, São Paulo 13561-901 Brazil

## Abstract

Combination of multifunctionalities into one compound is a rational strategy in medicinal chemical design, and have often been used with metallodrug-based compounds. In the present study, we synthesized a novel ruthenium-based 5-fluorouracil complex [Ru(5-FU)(PPh_3_)_2_(bipy)]PF_6_ (PPh_3_ = triphenylphosphine; and bipy = 2,2′-bipyridine) with enhanced cytotoxicity in different cancer cells, and assessed its apoptosis induction action in human colon carcinoma HCT116 cells. The complex was characterized by infrared, cyclic voltammetry, molar conductance measurements, elemental analysis, NMR experiments and X-ray crystallographic analysis. In both 2D and 3D cell culture models, the complex presented cytotoxicity to cancer cells more potent than 5-FU. A typical morphology of apoptotic cell death, increased internucleosomal DNA fragmentation, without cell membrane permeability, loss of the mitochondrial transmembrane potential, increased phosphatidylserine externalization and caspase-3 activation were observed in complex-treated HCT116 cells. Moreover, the pre-treatment with Z-DEVD-FMK, a caspase-3 inhibitor, reduced the apoptosis induced by the complex, indicating cell death by apoptosis through caspase-dependent and mitochondrial intrinsic pathways. The complex failed to induce reactive oxygen species production and DNA intercalation. In conclusion, the novel complex displays enhanced cytotoxicity to different cancer cells, and is able to induce caspase-mediated apoptosis in HCT116 cells.

## Introduction

Colon and rectal carcinoma (CRC) is the third most common type of cancer in the world^1^, and 5-fluorouracil (5-FU) is among the most common antineoplastic agent used in CRC treatment^2^. 5-FU-based chemotherapy is the first-line treatment for advanced CRC, but the response rates are about 10–15% with 5-FU as a monotherapy, and improve to only 40–50% when combined with irinotecan and oxaliplatin^3–7^. Therefore, new chemotherapy drugs for CRC are needed.

Ruthenium-based complexes are a potential novel class of antineoplastic chemotherapy that are currently under preclinical and phase I or II clinical trials^8–12^. Moreover, combination of multifunctionalities into one compound is a rational strategy in medicinal chemistry design, and have been often used with metallodrug-based compounds. Ruthenium complexes containing the η^6^-*p*-cymene ligand and 5-fluorouracil-1-methyl isonicotinate were previously prepared and tested in cancer cell lines; however, only complexes with less cytotoxicity than 5-FU were obtained^13^. Moreover, two ruthenium complexes containing 2-(5-fluorouracil)-yl-N-(pyridyl)-acetamide were synthesized, and exhibited DNA intercalation binding activity, but no cell-based assay was performed^14^. In this present paper, we synthesized a novel ruthenium-based 5-fluorouracil complex [Ru(5-FU)(PPh_3_)_2_(bipy)]PF_6_ (PPh_3_ = triphenylphosphine; and bipy = 2,2′-bipyridine) with enhanced cytotoxicity in different cancer cells, and its apoptosis induction was assessed in human colon carcinoma HCT116 cells.

## Results

### Synthesis of the complex [Ru(5-FU)(PPh_3_)_2_(bipy)]PF_6_

**Figure 1:**
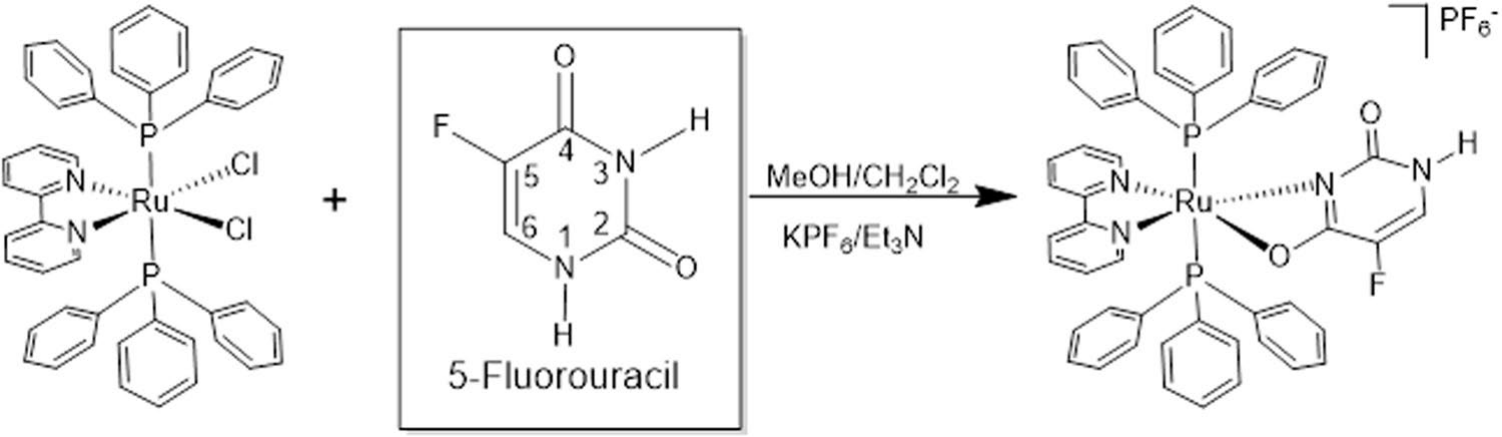
Synthetic route to obtain the complex [Ru(5-FU)(PPh_3_)_2_(bipy)]PF_6_.

The novel complex [Ru(5-FU)(PPh_3_)_2_(bipy)]PF_6_ was obtained from the precursor [RuCl_2_(PPh_3_)_2_(bipy)] by exchange of two chlorido ligands and coordination of one 5-FU as bidentate ligand. As shown in Fig. 1, the synthetic route of the ruthenium-based complex is straightforward, has provided very good yields and analytically pure complex, as determined by elemental analysis. The compound was characterized by spectroscopic and electrochemical techniques, and its crystal structure has been determined by single-crystal X-ray diffraction that confirmed its composition and stereochemistry. Also, the complex showed to be monocharged, presenting conductivity, in CH_2_Cl_2_, of 54.4 μS/cm, and thus, it is necessary a PF_6_^−^ as a counter-ion.

Infrared (IR) spectra and main bands assignments of the complex [Ru(5-FU)(PPh_3_)_2_(bipy)]PF_6_ and metal-free 5-FU confirmed the presence of the 5-FU ligand coordinated to the metal center (Supplementary Figure 1 and Supplementary Table 1). The IR spectrum of the complex shows broad absorptions at around 3173/cm occurring due to NH stretching vibration of 5-FU ligand, while in the region of 3078-2960/cm it is observed bands related to *v*C-H stretching vibrations of the aromatic groups of the PPh_3_, bipy and 5-FU. An interesting aspect observed in the fourier-transform-IR experiment is that in metal complex only one carbonyl group of 5-FU is coordinated to metal. The ligand free presents two *v*C=O bands occurring at 1735 and 1700/cm, while the complex presents only one *v*C=O stretching vibration band at 1659/cm. Also, strong bands in the range of 1606-1600 cm^−1^ can be attributed to *v*C=N, while at 1537-1481/cm can be assigned to vC=C stretching vibrations. The bands related to C-N group of six-membered rings of 5-FU occurs at around 1300-1270/cm. The coordination of 5-FU can be also confirmed by the *v*C-F stretching vibration resulting in a well-defined band at 1236/cm. Finally, the vRu-P, vRu-O and vRu-N stretching vibrations were assigned to weak bands in a region of low intensity of IR spectra at about 519, 496 and 464-403/cm, respectively. Electronic spectra of the complex show a broad band at around 296 nm assigned to an intraligand band, while the one at 420 nm can be assigned to charge transfer from Ru(II) to the ligands.

The redox behavior of the complex [Ru(5-FU)(PPh_3_)_2_(bipy)]PF_6_ was performed with potential range from 0 to +1.6 V, with a Pt disc electrode versus an Ag/AgCl reference electrode. The cyclic voltammogram exhibits a one-electron wave for Ru(II)/Ru(III) redox couple, with anodic peak, E_pa_, at +1370 mV (Supplementary Figure 2). The half-wave potential (E½) value for the complex (1308 mV) was more anodic than the precursor [RuCl_2_(PPh_3_)_2_(bipy)] that presents value of approximately 600 mV. This indicates that ruthenium is more easily oxidized in the precursor [RuCl_2_(PPh_3_)_2_(bipy)] than in the complex [Ru(5-FU)(PPh_3_)_2_(bipy)]PF_6_. As a result, the complex is more stable, and this stabilization is possible due to the replacement of two σ- and π-donor chlorides by a 5-FU monoanionic chelating ligand, which contains an N-acceptor group, which can act as σ-donor and π-acceptor.

The stereochemistry around metal can be also highlighted by the ^31^P{^1^H} nuclear magnetic resonance (NMR) spectrum of the complex [Ru(5-FU)(PPh_3_)_2_(bipy)]PF_6_ (Supplementary Figure 3). The presence of a singlet signal at 35.8 ppm indicates the magnetic equivalence of the two phosphorus atoms of PPh_3_ ligand, in which P is *trans* positioned to P. Recently, this same behavior was also observed in previous report with phosphorus *trans* to phosphorus^15^. The presence of the PF_6_^−^ counter-ion can be also confirmed by the heptet signal at around −144 ppm. In the ^1^H MNR experiments, the coordination of 5-FU can be also confirmed due to the presence of ligand signals at 10.4 and 7.8 ppm assigned to protons of the N1-H and C6-H groups, respectively (Supplementary Figure 4). In addition, in the region of 7.4–7.2 ppm, the 30 hydrogen attributed to two PPh_3_ ligand was confirmed.

**Figure 2:**
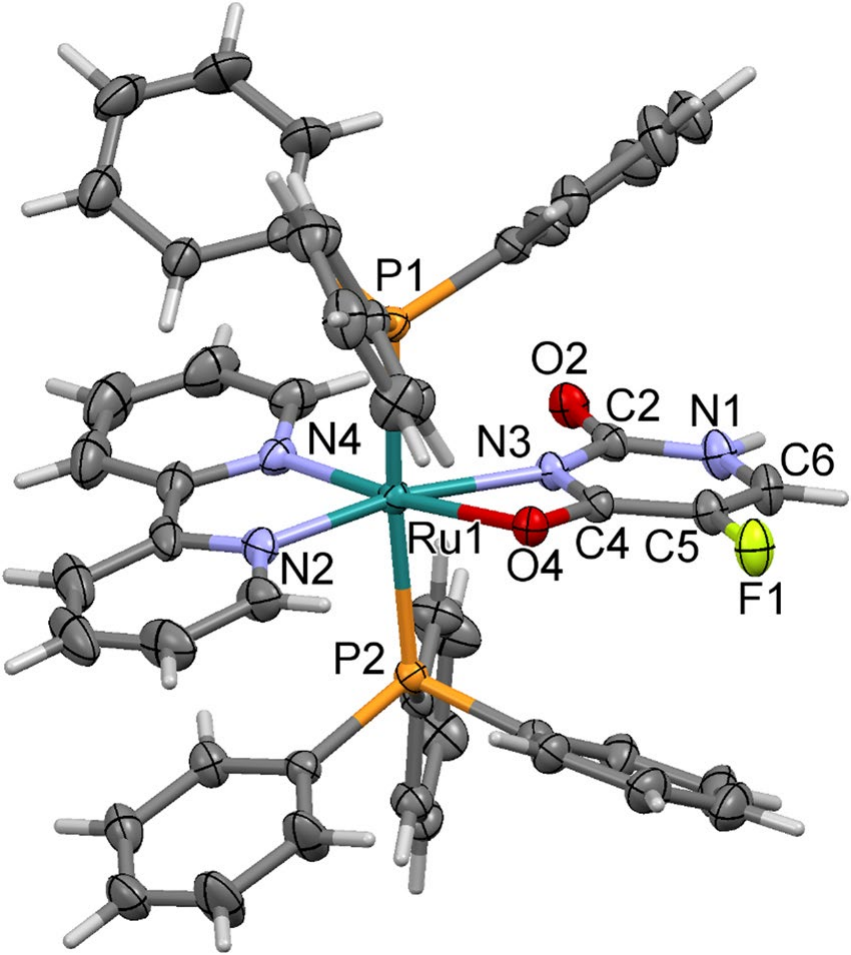
Crystal structure of the complex [Ru(5-FU)(PPh_3_)_2_(bipy)]PF_6_ with main atoms labelled and ellipsoids at 30% probability. For clarity, the PF_6_^−^ was omitted.

The crystal structure of the complex [Ru(5-FU)(PPh_3_)_2_(bipy)]PF_6_ is depicted in Fig. 2. It should be emphasized that this represent the first report of crystal structure of a ruthenium-based 5-fluorouracil complex. Crystal data collection and structure refinement parameters are summarized in Supplementary Table 2. The complex crystalizes in the P2_1_/n space group with one molecule of the complex and one disordered PF_6_^−^ anion in the asymmetric unit. The structure shows a distorted octahedral geometry such as observed by bond angles around the ruthenim center (Supplementary Table 3). The crystallographic analysis revels that the 5-FU ligand is coordinated to ruthenium as bidentate by N3 and O4 atoms.

Comparing molecular geometry of the complex [Ru(5-FU)(PPh_3_)_2_(bipy)]PF_6_ with the metal-free 5-FU^16^, it is observed that the coordination to Ru leads to small variation in the C-N, C-F and C=O bond lengths with atoms no involved in the coordination. In metal-free 5-FU, the N1-C2 and N1-C6 bond length are ranging 1.35–1.39 Å, and the in the complex the N1-C2 and N1-C6 bond length agree with these values (1.372 and 1.352 Å). In the complex, the C-F bond length is 1.347 Å, while in the metal-free 5-FU the value is close to 1.35 Å. As a result of ligand coordination, the bonds near to metal center present slight changes. In the complex, C4-O4 and C4-N3 bonds present values of 1.272 and 1.350 Å, respectively, while in the metal-free 5-FU the values found to these bonds are 1.24 and 1.39 Å. When 5-FU is coordinated to Ru(II) the length of these bonds changes significantly in which the C4–O4 is longer, whereas C4–N3 is shorter. This suggests that the molecule presents an electron delocalization on the [O4–C4–N3–Ru1] moiety, giving stabilization to the chelating system. The metal-free 5-FU and coordinated to Ru presents a planar conformation. In the complex, six-membered rings of 5-FU, bipy and PPh_3_ are stacked to form intramolecular π-π interactions with the adjacent ligands, stabilizing the molecular structure of the complex (Supplementary Figure 5).

The crystal packing of the complex [Ru(5-FU)(PPh_3_)_2_(bipy)]PF_6_ is stabilized mainly by well orientated hydrogen bonds, involving the N1–H1…O2 atoms [H…O distance of 1.916 Å and N…O separation of 2.773 Å] that form centrosymmetric dimmers (Supplementary Figure 6).

The high resolution mass spectrum of complex [Ru(5-FU)(PPh_3_)_2_(bipy)]PF_6_ is presented in the Supplementary Figure 7.

### The complex [Ru(5-FU)(PPh_3_)_2_(bipy)]PF_6_ displays enhanced cytotoxicity to different cancer cells

The cytotoxicity of the complex [Ru(5-FU)(PPh_3_)_2_(bipy)]PF_6_ was evaluated in cancer cell lines with different histological types (MCF7, HCT116, HepG2, SCC-9, HSC-3, HL-60, K-562 and B16-F10) and in two non-cancer cells (MRC-5 and PBMC) in two-dimensional (2D) culture by alamar blue assay 72 h after incubation, as shown in Table 1. The complex presented enhanced cytotoxicity to different cancer cells, with half maximal inhibitory concentration (IC_50_) values ranging from 1.5 to 7.6 μM for cancer cell lines HCT116 and MCF7, respectively. On the other hand, 5-FU presented IC_50_ values ranging from 1.3 to 127.1 μM for cancer cell lines HepG2 and SCC9, respectively. The complex was more potent than 5-FU in MCF7 (2-fold), HCT116 (3-fold), SCC9 (55-fold), HSC3 (10-fold), HL-60 (5-fold), K562 (10-fold) and B16-F10 (2-fold). Doxorubicin presented IC_50_ values ranging from 0.2 to 1.4 μM for cancer cell lines B16-F10 and MCF7, respectively. Oxaliplatin presented IC_50_ values ranging from 0.5 to 6.0 μM for cancer cell lines HL-60 and MCF7, respectively. The precursor of type [RuCl_2_(N-N)(P-P)] (N-N = diimines; P-P = diphosphines) had been previously tested and exhibited only weak cytotoxicity^17,18^, and was not tested in the present study.

**Table 1:** Cytotoxic activity of the complex [Ru(5-FU)(PPh_3_)_2_(bipy)]PF_6_ (RU/5-FU).

Cells	IC_50_ in µM			
	DOX	OXA	5-FU	RU/5-FU
MCF7	1.4	6.0	14.1	7.6
	0.6–2.4	4.0–10.0	11.3–18.1	3.8–15.4
HCT116	0.5	4.3	4.1	1.5
	0.1–0.7	2.8–6.1	1.9–8.5	1.3–1.8
HepG2	0.3	1.5	1.3	2.8
	0.1–0.8	0.8–2.4	0.3–6.1	2.1–3.8
SCC9	0.4	N.d	127.1	2.3
	0.3–0.9		117.0–143.3	1.7–3.0
HSC3	0.8	3.4	16.3	1.7
	0.3–1.2	1.5–5.4	11.3–19.5	1.4–2.0
HL-60	0.5	0.5	12.6	2.6
	0.2–0.9	0.1–2.2	9.5–16.7	2.3–2.9
K562	0.2	1.2	19.4	2.0
	0.1 - 0.6	0.5–2.5	12.7–24.7	1.7–2.5
B16-F10	0.2	0.7	3.5	2.1
	0.1–0.4	0.1–1.4	2.1–5.9	1.8–2.4
MRC-5	1.6	1.4	57.9	9.5
	1.1–2.3	0.7–2.2	39.8–84.4	8.3–10.9
PBMC	5.1	12.4	78.0	5.7
	2.3–8.8	8.5–17.4	66.4–84.1	4.1–7.4

Data are presented as IC_50_ values in μM and their respective 95% confidence interval obtained by nonlinear regression from at the least three independent experiments performed in duplicate, measured by alamar blue assay after 72 h incubation. Cancer cells: MCF7 (human breast carcinoma); HCT116 (human colon carcinoma); HepG2 (human hepatocellular carcinoma); SCC-9 (human oral squamous cell carcinoma); HSC-3 (human oral squamous cell carcinoma); HL-60 (human promyelocytic leukemia); K-562 (human chronic myelogenous leukemia); and B16-F10 (mouse melanoma). Non-cancer cells: MRC-5 (human lung fibroblast) and PBMC (human peripheral blood mononuclear cells). Doxorubicin (DOX), oxaliplatin (OXA) and 5-fluorouracil (5-FU) were used as the positive controls. N.d. Not determined.

The IC_50_ value for non-cancer cells was 9.5 and 5.7 μM for the complex and 57.9 and 78.0 μM for 5-FU in MRC5 and PBMC cells, respectively. In addition, the IC_50_ value in non-cancer cells was 1.6 and 5.1 μM for doxorubicin, 1.4 and 12.4 μM for oxaliplatin in MRC5 and PBMC cells, respectively. Table 2 shows the calculated selectivity index (SI). The complex exhibited SI similar to 5-FU, doxorubicin and oxaliplatin to most of the cell lines tested.

**Table 2:** Selectivity index of the complex [Ru(5-FU)(PPh_3_)_2_(bipy)]PF_6_ (RU/5-FU).

Cancer cells	Non-cancer cells							
	MRC-5				PBMC			
	DOX	OXA	5-FU	RU/5-FU	DOX	OXA	5-FU	RU/5-FU
MCF7	1.1	0.2	4.1	1.3	3.6	2	5.5	0.8
HCT116	3.2	0.3	14.1	6.3	10.2	2.9	19	3.8
HepG2	5.3	0.9	44.5	3.4	17	8.3	60	2.1
SCC-9	4	N.d.	0.5	4.1	12.8	N.d.	0.6	2.5
HSC-3	2	0.4	3.6	5.6	6.4	3.7	4.8	3.4
HL-60	3.2	2.8	4.6	3.7	10.2	24.8	6.2	2.2
K-562	8	1.2	3	4.8	25.5	10.3	4	2.9
B16-F10	8	2	16.5	4.5	25.5	17.7	22.3	2.7

Data are presented the selectivity index (SI) calculated using the following formula: SI = IC_50_[non-cancer cells]/IC_50_[cancer cells]. Cancer cells: MCF7 (human breast carcinoma); HCT116 (human colon carcinoma); HepG2 (human hepatocellular carcinoma); SCC-9 (human oral squamous cell carcinoma); HSC-3 (human oral squamous cell carcinoma); HL-60 (human promyelocytic leukemia); K-562 (human chronic myelogenous leukemia); and B16-F10 (mouse melanoma). Non-cancer cells: MRC-5 (human lung fibroblast) and PBMC (human peripheral blood mononuclear cells). Doxorubicin (DOX), oxaliplatin (OXA) and 5-fluorouracil (5-FU) were used as the positive controls. N.d. Not determined.

**Figure 3:**
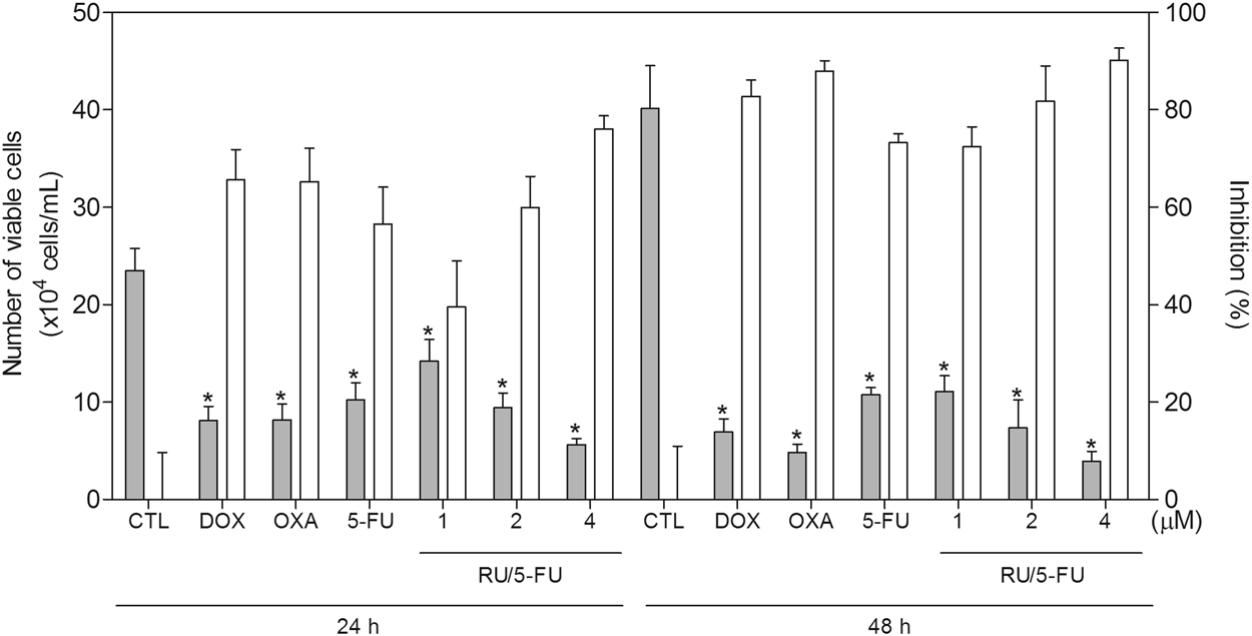
Effect of the complex [Ru(5-FU)(PPh_3_)_2_(bipy)]PF_6_ (RU/5-FU) in the cell viability of HCT116 cells determined by trypan blue staining. The gray bars represent number of viable cells (x10^4^cells/mL) and the white bars represent cell inhibition (%). The negative control (CTL) was treated with the vehicle (0.1% DMSO) used for diluting the compound tested. Doxorubicin (DOX, 1 µM), oxaliplatin (OXA, 2.5 µM) and 5-fluorouracil (5-FU, 4 µM) were used as the positive controls. Data are presented as the mean ± S.E.M. of three independent experiments performed in duplicate. *P < 0.05 compared with the negative control by ANOVA followed by Student Newman-Keuls test.

HCT116 cell line was the most sensitive cell line to the complex [Ru(5-FU)(PPh_3_)_2_(bipy)]PF_6_ and was used as a cellular model to the next experiments. Therefore, the cytotoxicity of the complex was confirmed by trypan blue exclusion (TBE) assay in HCT116 cells, after 24 and 48 h incubation. The complex significantly reduced (*p* < 0.05) the number of viable cells (Fig. 3). At concentrations of 1, 2 and 4 μM, the complex reduced the number of viable cells by 40.0, 60.0 and 76.1% after 24 h, and 72.4, 81.7 and 90.2% after 48 h, respectively. 5-FU, at 4 μM, reduced the number of viable cells by 56.6 after 24 h, and 73.2 after 48 h. No significant (*p* > 0.05) increase in the number of non-viable cells was observed. Doxorubicin and oxaliplatin also reduced the number of viable cells after 24 and 48 h of incubation.

**Figure 4:**
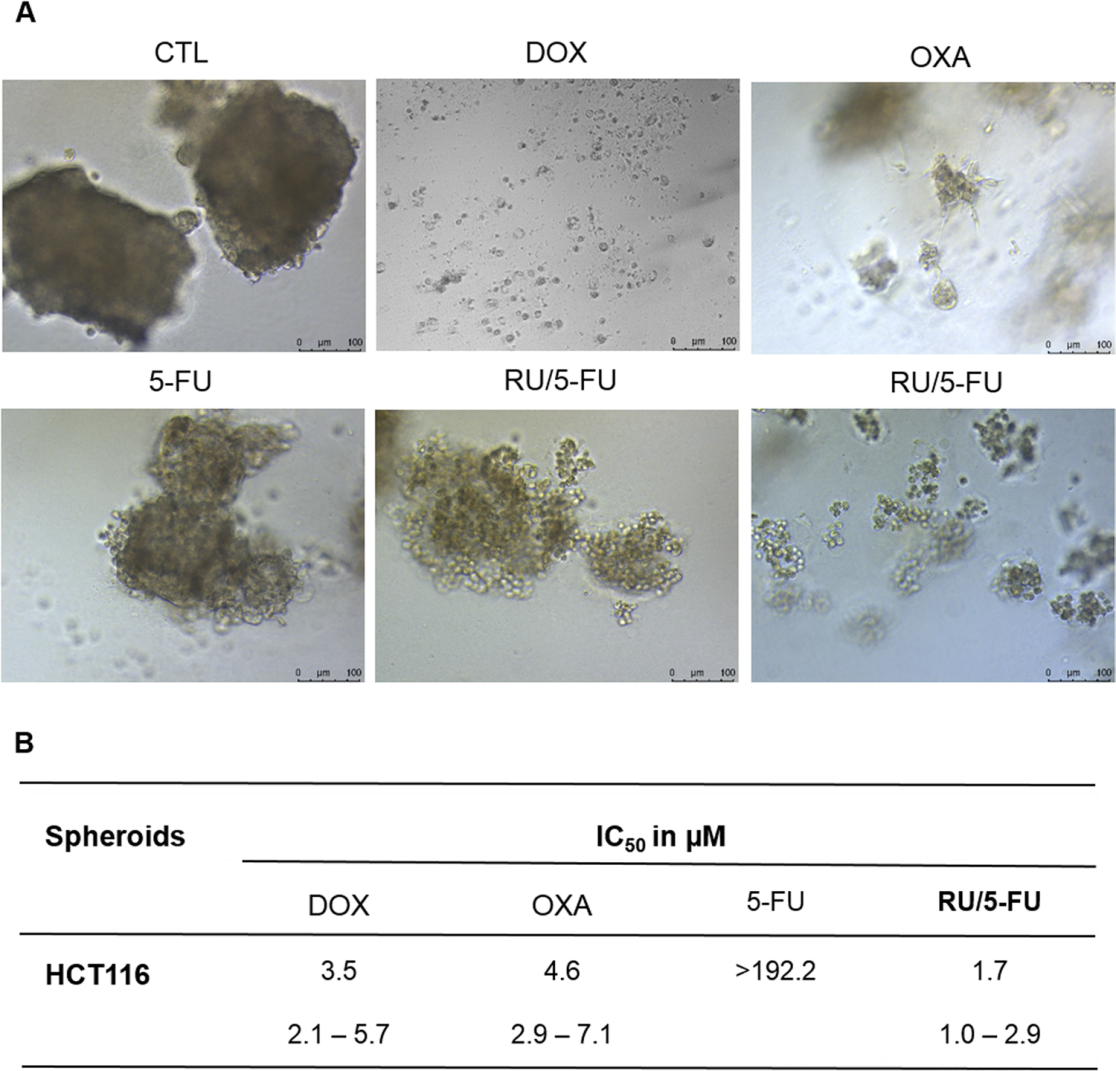
Effect of the complex [Ru(5-FU)(PPh_3_)_2_(bipy)]PF_6_ (RU/5-FU) in 3D *in vitro* model of cancer multicellular spheroids formed from HCT116 cells. (**A**) Cells examined by light microscopy (bar = 100 µm). (**B**) IC_50_ values in μM and their respective 95% confidence interval obtained by nonlinear regression from three independent experiments performed in duplicate, measured by alamar blue assay 72 h after incubation. The negative control (CTL) was treated with the vehicle (0.1% DMSO) used for diluting the compound tested. Doxorubicin (DOX), oxaliplatin (OXA) and 5-fluorouracil (5-FU) were used as the positive controls.

The cytotoxicity of the complex [Ru(5-FU)(PPh_3_)_2_(bipy)]PF_6_ was also performed in an *in vitro* three-dimensional (3D) model of cancer multicellular spheroids formed from HCT116 cells. The treatment with the complex disrupted these cell aggregations, resulting in the presence of cell debris (Fig. 4A). The IC_50_ of the complex was 1.7 μM (Fig. 4B), while 5-FU presented IC_50_ > 192.2 μM. The complex was more potent than 5-FU at the least 113-fold. Doxorubicin and oxaliplatin showed IC_50_ of 3.5 and 4.6 μM, respectively.

### Unlike 5-fluorouracil, the complex [Ru(5-FU)(PPh_3_)_2_(bipy)]PF_6_ does not induce S phase arrest

The cell cycle distribution in HCT116 cells treated with the complex [Ru(5-FU)(PPh_3_)_2_(bipy)]PF_6_ was assessed by DNA content using flow cytometry technique after 24 and 48 h incubation. Table 3 shows the obtained cell cycle distribution. All DNA that was sub-diploid in size (sub-G_0_/G_1_) was considered fragmented. The treatment with the complex caused a significant increase in the internucleosomal DNA fragmentation (*p* < 0.05) that became more pronounced at highest concentration and at longest time of incubation. No accumulation of cells in any phase of the cell cycle was observed. On the other hand, 5-FU treatment resulted in a significantly increase in the number of cells in S phase compared to the negative control (30.7% at control against 54.0% at 5-FU after 24 h incubation; and 23.8% at control against 40.8% at 5-FU after 48 h incubation, respectively). Doxorubicin and oxaliplatin caused cell cycle arrest at the phase G_2_/M in HCT116 cells.

**Table 3:** Effect of the complex [Ru(5-FU)(PPh_3_)_2_(bipy)]PF_6_ (RU/5-FU) in the cell cycle distribution of HCT116 cells.

Treatment	Concentration (µM)	DNA content (%)			
		Sub-G_0_/G_1_	G_0_/G_1_	S	G_2_/M
**24** **h incubation**					
CTL	—	3.9 ± 1.0	42.0 ± 2.5	12.6 ± 2.8	28.1 ± 5.5
DOX	1	9.7 ± 2.5	28.0 ± 6.3	10.0 ± 2.5	44.1 ± 3.4*
OXA	2.5	8.8 ± 3.5	32.4 ± 3.7	13.5 ± 3.1	41.6 ± 1.6*
5-FU	4	11.5 ± 4.6	37.7 ± 1.8	25.9 ± 3.4*	16.9 ± 1.1
RU/5-FU	1	8.8 ± 1.8	43.6 ± 2.9	8.8 ± 2.3	32.1 ± 1.7
	2	15.4 ± 3.2	35.3 ± 2.0	14.5 ± 2.7	28.0 ± 3.0
	4	25.9 ± 5.1*	19.9 ± 3.6*	15.1 ± 2.3	27.0 ± 2.5
**48** **h incubation**					
CTL	—	3.3 ± 0.7	44.8 ± 1.1	13.5 ± 2.4	26.0 ± 2.3
DOX	1	18.3 ± 2.5	22.5 ± 4.0*	14.9 ± 2.5	44.2 ± 4.4*
OXA	2.5	17.7 ± 1.9	36.2 ± 2.6	7.6 ± 0.4	38.0 ± 3.4
5-FU	4	18.1 ± 3.0	28.9 ± 3.9*	31.8 ± 1.5*	17.3 ± 1.2
RU/5-FU	1	21.8 ± 4.7*	34.5 ± 2.9	14.1 ± 1.6	18.8 ± 2.4
	2	28.9 ± 4.7*	31.8 ± 2.3*	9.3 ± 1.7	21.2 ± 2.8
	4	55.3 ± 2.7*	15.1 ± 0.9*	6.9 ± 1.3	17.1 ± 2.2

Data are presented as the mean ± S.E.M. of three independent experiments performed in duplicate. The negative control (CTL) was treated with the vehicle (0.1% DMSO) used for diluting the compound tested. Doxorubicin (DOX), oxaliplatin (OXA) and 5-fluorouracil (5-FU) were used as the positive controls. Ten thousand events were evaluated per experiment and cellular debris was omitted from the analysis. *P < 0.05 compared with the negative control by ANOVA followed by Student Newman-Keuls Test.

### The complex [Ru(5-FU)(PPh_3_)_2_(bipy)]PF_6_ induces apoptosis through caspase-dependent and mitochondrial intrinsic pathway in HCT116 cells

**Figure 5:**
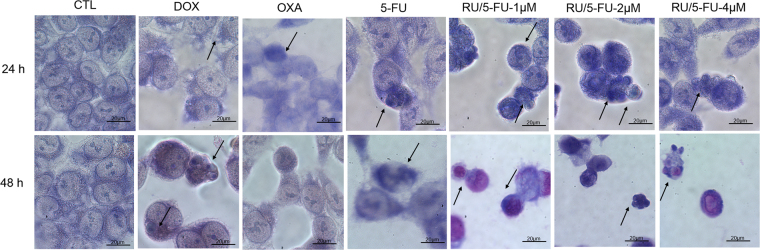
Effect of the complex [Ru(5-FU)(PPh_3_)_2_(bipy)]PF_6_ (RU/5-FU) in the morphological analysis of HCT116 cells after 24 and 48 h incubation. The cells were stained with may-grunwald-giemsa and examined by light microscopy (bar = 20 µm). Arrows indicated cells with reduction in the cell volume, chromatin condensation or fragmented DNA. The negative control (CTL) was treated with the vehicle (0.1% DMSO) used for diluting the compound tested. Doxorubicin (DOX, 1 µM), oxaliplatin (OXA, 2.5 µM) and 5-fluorouracil (5-FU, 4 µM) were used as positive controls.

As observed by May-Grunwald-Giemsa staining, the treatment with the complex induced cell shrinkage and fragmentation of the nuclei of HCT116 cells (Fig. 5). Doxorubicin, oxaliplatin and 5-FU also induced cell shrinkage and nuclear fragmentation.

**Figure 6:**
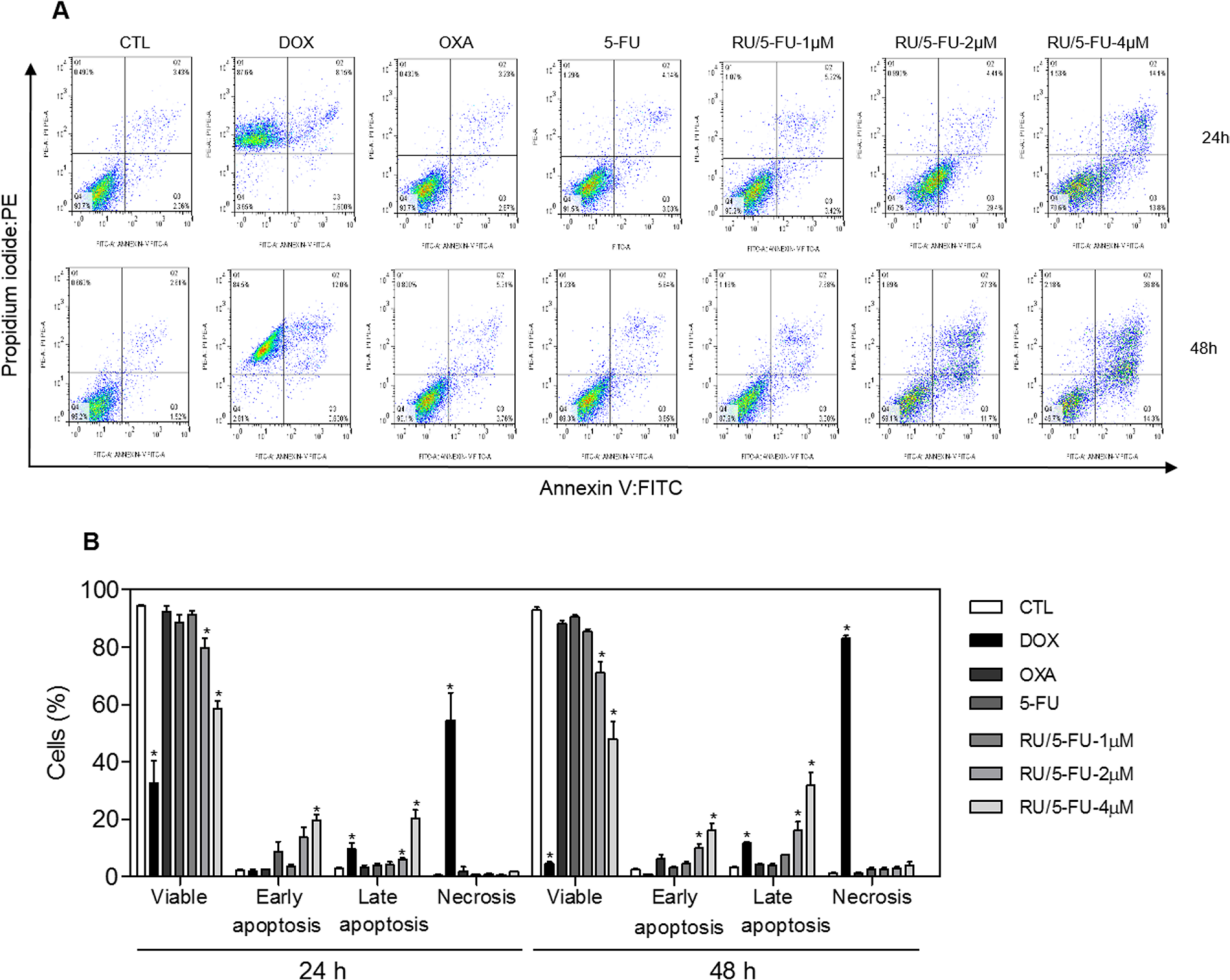
Effect of the complex [Ru(5-FU)(PPh_3_)_2_(bipy)]PF_6_ (RU/5-FU) in the apoptosis induction in HCT116 cells determined by flow cytometry using annexin V-FITC/PI staining 24 and 48 h after incubation. (**A**) Representative flow cytometry dot plots showing the percentage of cells in viable, early apoptotic, late apoptotic and necrotic stages. (**B**) Quantification of the cell viability. The negative control (CTL) was treated with the vehicle (0.1% DMSO) used for diluting the compound tested. Doxorubicin (DOX, 1 µM), oxaliplatin (OXA, 2.5 µM) and 5-fluorouracil (5-FU, 4 µM) were used as the positive controls. Data are presented as the mean ± S.E.M. of three independent experiments performed in duplicate. Ten thousand events were evaluated per experiment and cellular debris was omitted from the analysis. *p < 0.05 compared with the negative control by ANOVA followed by Student Newman-Keuls test.

**Figure 7:**
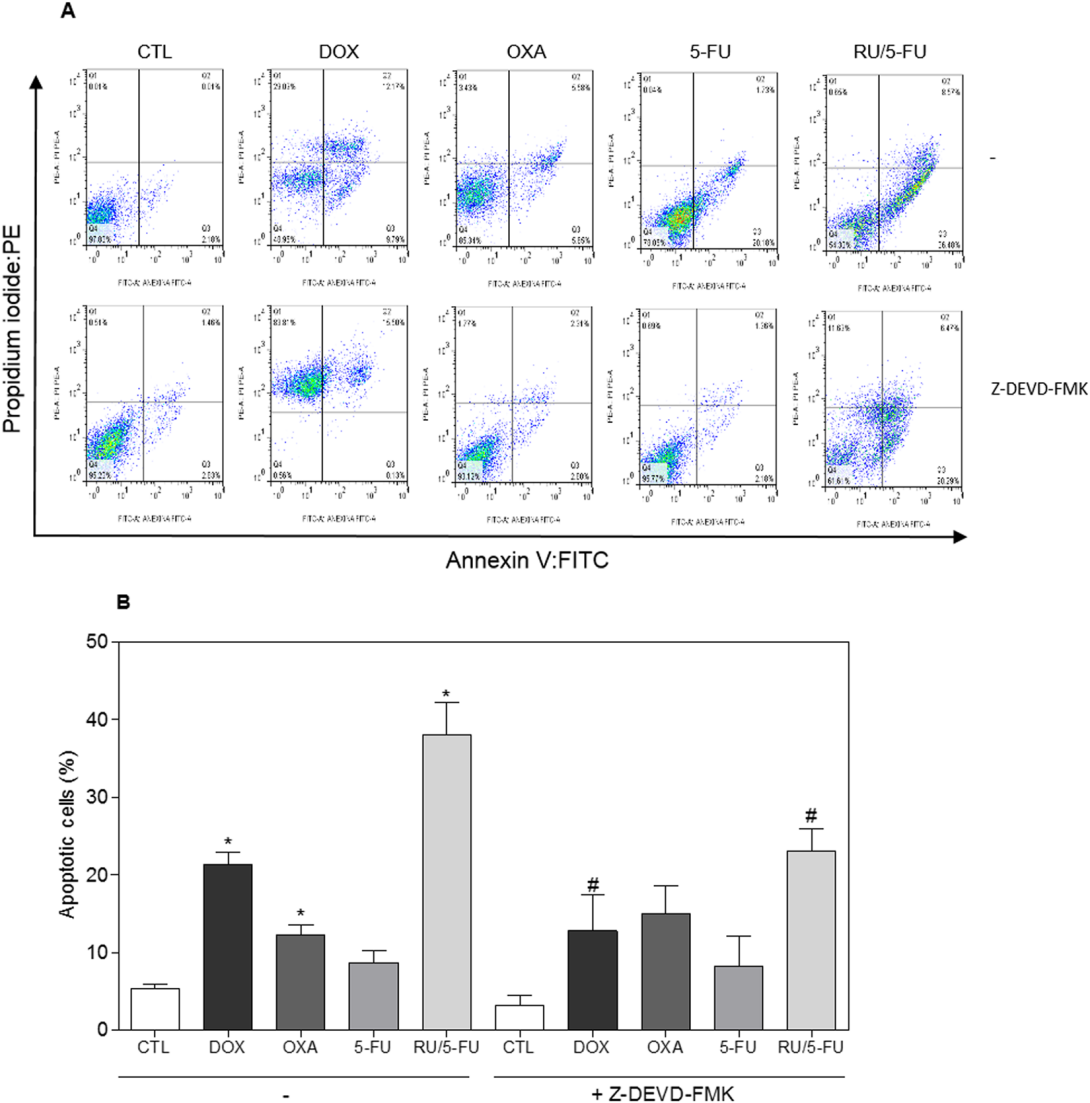
Effect of the caspase-3 inhibitor (Z-DEVD-FMK) in the apoptosis induced by the complex [Ru(5-FU)(PPh_3_)_2_(bipy)]PF_6_ (RU/5-FU) on HCT116 cells determined by flow cytometry using annexin V-FITC/PI staining. (**A**) Representative flow cytometric dot plots showing the percentage of cells in viable, early apoptotic, late apoptotic and necrotic stages. (**B**) Quantification of apoptotic cells. The cells were pre-treated for 2 h with 50 µM Z-DEVD-FMK, then incubated with 4 μM RU/5-FU for 48 h. The negative control (CTL) was treated with the vehicle (0.1% DMSO) used for diluting the compound tested. Doxorubicin (DOX, 1 µM), oxaliplatin (OXA, 2.5 µM) and 5-fluorouracil (5-FU, 4 µM) were used as the positive controls. Data are presented as the mean ± S.E.M. of three independent experiments performed in duplicate. Ten thousand events were evaluated per experiment and cellular debris was omitted from the analysis. *p < 0.05 compared with the negative control by ANOVA followed by Student Newman-Keuls test. ^#^ *p* < 0.05 compared with the respective treatment without inhibitor by ANOVA followed by Student Newman-Keuls test.

**Figure 8:**
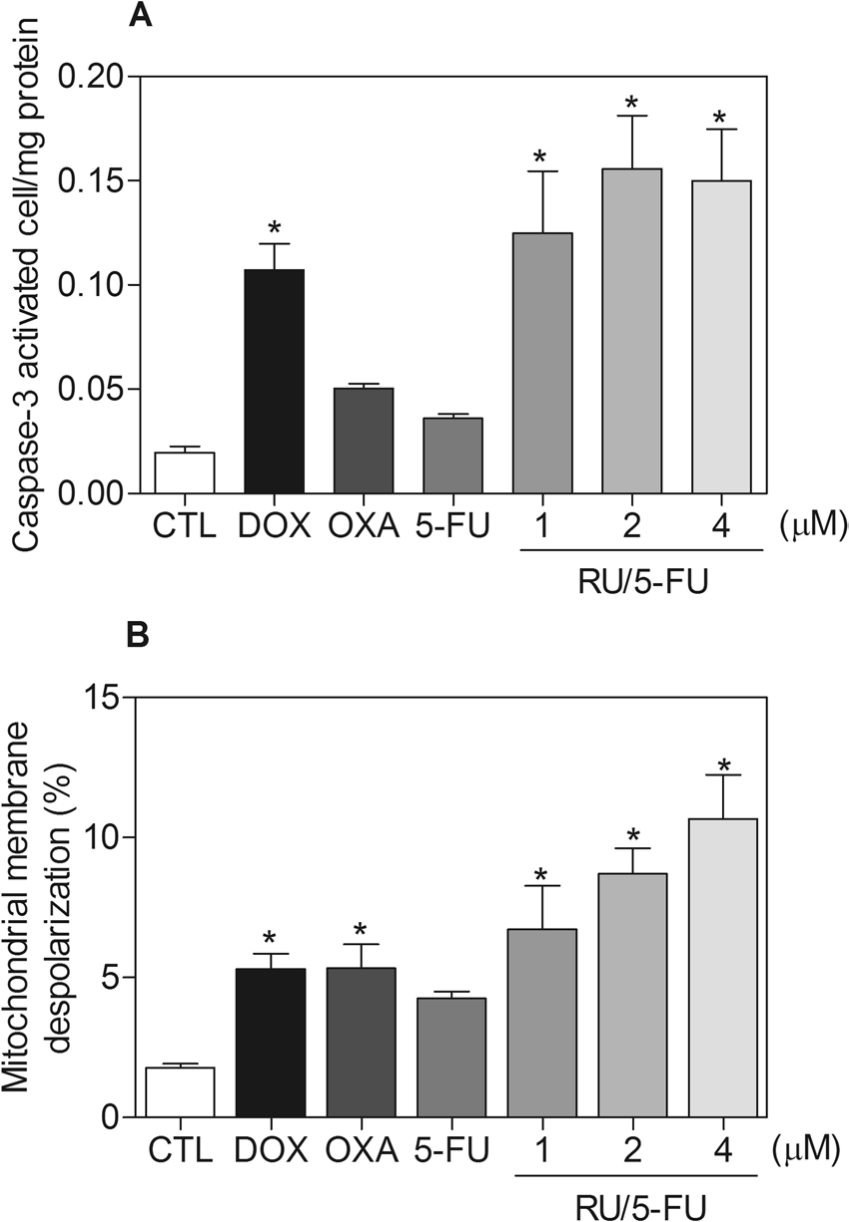
Effect of the complex [Ru(5-FU)(PPh_3_)_2_(bipy)]PF_6_ (RU/5-FU) in the caspase-3 activity and mitochondrial membrane potential on HCT116 cells. (**A**) Caspase-3 activity determined by colorimetric assay 48 h after incubation. (**B**) Mitochondrial membrane potential determined by flow cytometry using rhodamine 123 staining 24 h after incubation. The negative control (CTL) was treated with the vehicle (0.1% DMSO) used for diluting the compound tested. Doxorubicin (DOX, 1 µM), oxaliplatin (OXA, 2.5 µM) and 5-fluorouracil (5-FU, 4 µM) were used as the positive controls. Data are presented as the mean ± S.E.M. of three independent experiments performed in duplicate. Ten thousand events were evaluated per experiment and cellular debris was omitted from the analysis. *p < 0.05 compared with the negative control by ANOVA followed by Student Newman-Keuls test.

Annexin V-FITC and propidium iodide (PI) double staining was performed by flow cytometry to measure the percentage of cells in viable, early apoptotic, late apoptotic and necrotic stages. The treatment with the complex [Ru(5-FU)(PPh_3_)_2_(bipy)]PF_6_ resulted in increasing in early and late apoptotic cells in a time- and concentration-dependent manners (Fig. 6A and B). No significant increase in necrotic cells was observed in HCT116 cells treated with the complex. In addition, pre-incubation with a caspase-3 inhibitor, Z-DEVD-FMK, prevented the increase of apoptotic cells caused by the complex (Fig. 7A and B). A significant increase in caspase-3 activation was also observed in HCT116 cells treated with the complex, as measured by colorimetric assay using DEVD-pNA as the substrate (Fig. 8A). Moreover, the mitochondrial membrane potential was measured by the incorporation of rhodamine 123 using flow cytometry, and the treatment with the complex also induced mitochondrial depolarization in HCT116 cells (Fig. 8B). At the concentration tested (4 μM, based on its IC_50_ value), 5-FU did not induce increasing in the early and late apoptosis, suggesting that 5-FU has only anti-proliferative action. Mitochondrial depolarization and activation of caspase-3 were also not affected in 5-FU-treated HCT116 cells, at the concentration tested.

The cytotoxicity of the complex [Ru(5-FU)(PPh_3_)_2_(bipy)]PF_6_ in BAD KO SV40 MEF (BAD gene knockout immortalized mouse embryonic fibroblast) and its parental cell line WT SV40 MEF (wild-type immortalized mouse embryonic fibroblast) was also evaluated by alamar blue assay after 72 h incubation. The IC_50_ values for the complex was 1.8 and 1.9 μM for BAD KO SV40 MEF and WT SV40 MEF cell lines, respectively, indicating that BAD gene is not essential for its cytotoxic activity. 5-FU showed IC_50_ values of 7.3 and 1.7 μM, while doxorubicin showed IC_50_ values of 0.4 and 0.03 μM on BAD KO SV40 MEF and WT SV40 MEF cell lines, respectively.

The action of the complex [Ru(5-FU)(PPh_3_)_2_(bipy)]PF_6_ in the intracellular reactive oxygen species (ROS) levels was evaluated in HCT116 cells by flow cytometry using the redox-sensitive fluorescent probe 2′,7′-dichlorofluorescin diacetate (DCF-DA). The complex did not induce significant increase in ROS levels after 1 or 3 h incubation (data not shown). In addition, pre-treatment with the antioxidant N-acetyl-L-cysteine (NAC) did not prevent the increase of apoptotic cells caused by the complex (data not shown).

### The complex [Ru(5-FU)(PPh_3_)_2_(bipy)]PF_6_ does not induce DNA intercalation

DNA intercalation was evaluated by examining the ability of the complex [Ru(5-FU)(PPh_3_)_2_(bipy)]PF_6_ to displace ethidium bromide from calf thymus DNA (ctDNA). In this assay, the fluorescence intensity of ethidium bromide decreases if the compound test displaces ethidium bromide from DNA. However, the complex did not decrease the ethidium bromide fluorescence, indicating that it is not a strong DNA intercalator. Doxorubicin, a known DNA intercalator, reduced the fluorescence intensity of ethidium bromide (data not shown).

## Discussion

5-FU is an important chemotherapeutic drug widely used in cancer treatment, and ruthenium-based complexes have been shown as potent cytotoxic agents to cancer cells, possibly becoming as a new class of chemotherapeutic drugs. Herein, we combined these two components into one compounds and found a novel ruthenium-based 5-fluorouracil complex with enhanced cytotoxicity.

As mentioned above, some ruthenium complexes containing 5-FU as ligand were previously synthesized, but with weak cytotoxicity^13,14^. These ruthenium-based compounds were synthesized using precursor of type [Ru(*η* ^6^-arene)]. In this present paper, we used precursor of type [RuCl_2_(N-N)(P-P)] and obtained the complex [Ru(5-FU)(PPh_3_)_2_(bipy)]PF_6_, a compound with cytotoxicity to cancer cells more potent than 5-FU in both 2D and 3D cell culture models. The SI of the complex was similar to 5-FU, doxorubicin and oxaliplatin to most of the cell lines tested. In fact, the precursor of type [RuCl_2_(N-N)(P-P)] has been previously used by us to synthesize potent cytotoxic agents^12,18^.

5-FU is an antimetabolite drug that is incorporated into the DNA and RNA, and inhibits the enzyme thymidylate synthase. The incorporation of 5-FU into DNA and RNA occurs mainly during the S phase of cell cycle. Moreover, the inhibition of thymidylate synthase can cause deoxynucleotide imbalance during DNA synthesis. Therefore, 5-FU acts targeting S phase of the cell cycle^6,19,20^. In fact, we observed that 5-FU block S phase of the cell cycle in HCT116 cells. However, although the complex [Ru(5-FU)(PPh_3_)_2_(bipy)]PF_6_ increased significantly the internucleosomal DNA fragmentation, no accumulation of the cells in any phase of the cell cycle was observed, indicating different mechanism of action between 5-FU and its ruthenium-based complex. Many ruthenium complexes have been reported to cause block at different phases of the cell cycle. The ruthenium methylimidazole complex caused cell cycle arrest at G_0_/G_1_ phase and induced apoptosis via the mitochondrial pathway, which involved ROS accumulation, mitochondrial dysfunction and Bcl-2 and caspase activation in lung cancer A549 cells^21^. Three ruthenium(II)-arene complexes, namely [(*η* ^6^-*p*-cymene)Ru(Me_2_dppz)Cl]PF_6_, caused arrest of the cell cycle in G_2_/M and S phases in cervical carcinoma HeLa cells^22^. The ruthenium(II) polypyridyl complexes induce cell cycle arrest at G_2_/M phase in hepatocellular carcinoma BEL-7402 cells^11^.

As observed by a typical morphology of apoptotic cell death, increased internucleosomal DNA fragmentation, without cell membrane permeability, loss of the mitochondrial transmembrane potential, increased phosphatidylserine externalization and caspase-3 activation, the complex [Ru(5-FU)(PPh_3_)_2_(bipy)]PF_6_ induced apoptosis through caspase-dependent and mitochondrial intrinsic pathway in HCT116 cells. 5-FU is a known pro-apoptotic chemotherapy drug^6,19,23,24^. In addition, ruthenium complexes are also apoptosis inductors, including the ruthenium complexes [Ru(Cl-Ph-tpy)(phen)Cl]Cl and [Ru(Cl-Ph-tpy)(o-bqdi)Cl]Cl that decreased Bcl-2/Bax ratio causing cytochrome c mitochondrial release, the activation of caspase-3 and induction of apoptosis in HeLa cells^25^.

As mentioned, two ruthenium complexes containing 2-(5-fluorouracil)-yl-N-(pyridyl)-acetamide exhibited DNA intercalation action^14^; however, the complex [Ru(5-FU)(PPh_3_)_2_(bipy)]PF_6_ failed to induce DNA intercalation.

In conclusion, the complex [Ru(5-FU)(PPh_3_)_2_(bipy)]PF_6_ was synthesized for the first time at this communication and tested against cancer cells with different histological type. In both 2D and 3D cell culture models, the complex presented cytotoxicity to cancer cells more potent than 5-FU. Unlike 5-FU, the complex does not induce S phase arrest, indicating different mechanism of action between 5-FU and its ruthenium-based complex. In addition, the complex was able to induce apoptosis through caspase-dependent and mitochondrial intrinsic pathway in HCT116 cells.

## Material and Methods

### Chemistry

#### General

Elemental analysis (C, H and N) was performed using an FISONS instrument, CHNS EA-1108. The FT-IR spectra of complex and metal-free 5-FU was recorded on a FT-IR Bomem-Michelson 102 spectrometer as CsI pellets in the range of 4000-200/cm. The electronic spectra on a Hewlett Packard diode array-8452A scanning spectrophotometer and conductivity value were obtained using a Meter Lab CDM2300 instrument at room temperature, using 10^−3^ M solutions of the complex, both experiments were carried out in CH_2_Cl_2_. Cyclic voltammetry measurements were carried out at room temperature in an electrochemical analyzer BAS, model 100B. These experiments were also performed using CH_2_Cl_2_ containing Bu_4_NClO_4_ (TBAP) (Fluka Purum) at concentration of 0.10 M, as a supporting electrolyte and a one-compartment electrochemical cell based on three electrodes: Pt foils as the working and auxiliary electrodes and an Ag/AgCl (0.10 M TBAP in CH_2_Cl_2_) as the reference electrode. Under these conditions, the ferrocene (Fc) is oxidized at +0.43 V (Fc+/Fc). High-resolution electrospray ionization mass spectrometry (HRESIMS) spectrum was measured by direct infusion in a MicroTof–Q II Bruker Daltonics Mass Spectrometer (Le) in the positive ion mode, employing methanol as solvent (LC/MS grade from Honeywell/B&J Brand). ^31^P{1 H} and ^1^H NMR spectra were recorded in acetone-d_6_, on a Bruker DRX, with the ^31^P{^1^H} and ^1^H chemical shifts reported in relation to H_3_PO_4_ (85%) and tetramethylsilane, respectively. Single-crystals of the complex were obtained from ethyl ether/dichloromethane solvent diffusion. X-ray diffraction experiment was carried out at room temperature on an Enraf–Nonius Kappa-CCD diffractometer with graphite monochromated MoKα radiation (λ = 0.71073 Å). The structure was solved by direct methods and refined by full-matrix least-squares method on F^2^ with anisotropic thermal parameters for all non-hydrogen atoms using SHELXS-97^26^. All hydrogen atoms were placed in calculated positions and refined isotropically. Representation of the structure was drawn with the program Mercury 3.8^27^.

#### Synthesis of the complex [Ru(5-FU)(PPh_3_)_2_(bipy)]PF_6_

In a Schlenk flask, 24 mg (0.18 mmol) of 5-FU, 20 mg of KPF_6_, and 20 µL triethylamine (et_3_N) were dissolved in 20 mL of methanol. After, 100 mg (0.104 mmol) of [RuCl_2_(PPh_3_)_2_(bipy)] precursor solubilized in 80 mL of CH_2_Cl_2_ was added to the solution. The mixture was kept under stirring at reflux for 24 h. Then, the volume reduced to *ca*. 2 mL and 10 mL of water was added. A orange solid was precipitated and separated by filtration, washed with water, diethyl ether and dried under vacuum to yield 105 mg (85%). Anal. Calc. for [RuC_50_H_40_F_7_O_2_N_4_P_3_]: exp. (calc) 56.73 (56.88); H, 3.84 (3.82); N, 5.15 (5.31) %. Molar conductance (S.cm^2^.mol^−1^, CH_2_Cl_2_) 54.4. IR (cm^−1^): 3173, 3078, 3055, 3022, 2960, 1659, 1606, 1600, 1537, 1481, 1435, 1329, 1300, 1270, 1236, 1188, 1161, 1092, 1028, 999, 845, 766, 746, 698, 617, 557, 519, 496, 464, 403. HRESIMS (methanol), *m/z*: 911.1658 [M–PF_6_]^+^. ^31^P{^1^H} NMR (162 MHz, acetone-d_6_, 298 K): δ(ppm) 35.8 (s). UV–Vis (CH_2_Cl_2_, 1.6 × 10^−4^ M): λ/nm (ε/M^−1^cm^−1^) 330 (6704); 420 (3914).

### Biology action

#### Cells

MCF7 (human breast carcinoma), HCT116 (human colon carcinoma), HepG2 (human hepatocellular carcinoma), SCC-9 (human oral squamous cell carcinoma), HSC-3 (human oral squamous cell carcinoma), HL-60 (human promyelocytic leukemia), K-562 (human chronic myelogenous leukemia), B16-F10 (mouse melanoma), MRC-5 (human lung fibroblast), WT SV40 MEF (wild-type immortalized mouse embryonic fibroblast) and BAD KO SV40 MEF (BAD gene knockout immortalized mouse embryonic fibroblast) cell lines were obtained from the American Type Culture Collection (ATCC, Manassas, VA, USA). Cells were cultured in complete medium with appropriate supplements, as recommended by ATCC. All cell lines were tested for mycoplasma using a mycoplasma stain kit (Sigma-Aldrich Co., Saint Louis, MO, USA) to validate the use of cells free from contamination. Primary cell culture of peripheral blood mononuclear cells (PBMC) was obtained by standard ficoll density protocol. The Research Ethics Committee of the Oswaldo Cruz Foundation (Salvador, Bahia, Brazil) approved the experimental protocol (# 031019/2013). Cell viability was examined using TBE assay for all experiments.

#### Cytotoxic activity assay

Cell viability was quantified using the alamar blue assay, according to Ahmed *et al*.^28^. Cells were inserted in 96-well plates for all experiments (7 × 10^4^ cells/mL for adherent cells or 3 × 10^5^ cells/mL for suspended cells in 100 μL of medium). After 24 h, the complex was dissolved in dimethyl sulfoxide (DMSO, Sigma-Aldrich co.) and added to each well and incubated for 72 h. Doxorubicin (purity ≥ 95%, doxorubicin hydrochloride, Laboratory IMA S.A.I.C., Buenos Aires, Argentina), oxaliplatin (Sigma-Aldrich Co.) and 5-FU (Sigma-Aldrich Co.) were used as the positive controls. Four (for cell lines) or 24 h (for PBMCs) before the end of incubation, 20 μL of a stock solution (0.312 mg/mL) of the alamar blue (resazurin, Sigma-Aldrich Co.) were added to each well. Absorbance at 570 nm and 600 nm was measured using the SpectraMax 190 Microplate Reader (Molecular Devices, Sunnyvale, CA, EUA), and the drug effect was quantified as the percentage of control absorbance.

#### 3D multicellular spheroids culture

HCT116 cells were cultivated in 3D multicellular spheroids. Briefly, 100 μL of a solution of cells (0.5 × 10^6^ cells/mL) were inserted in 96-well plate with a cell-repellent surface (Greiner Bio-One; Kremsmünster, Austria) and cultured in complete medium plus 3% matrigel (BD Biosciences; San Jose, CA, EUA). Spheroids with stable structures had formed after three days. Then, the spheroids were exposed to a range of drug concentrations for 72 h, after which the cell viability was quantified by alamar blue assay as described above.

#### Trypan blue exclusion assay

TBE assay was used to confime the cytotoxic effect of the complex tested. The number of viable cells and non-viable cells (take up trypan blue) were counted. Briefly, 90 μL was removed from the cell suspension and 10 μL of trypan blue (0.4%) was added. Cell counting was performed using a light microscope with a hemocytometer filled with an aliquot of the homogenized cell suspension.

#### Internucleosomal DNA fragmentation and cell cycle distribution

Cells were harvested in a permeabilization solution containing 0.1% triton X-100, 2 µg/mL PI, 0.1% sodium citrate and 100 µg/mL RNAse (all from Sigma Chemical Co.) and incubated in the dark for 15 min at room temperature^29^. Finally, cell fluorescence was measured by flow cytometry on a BD LSRFortessa cytometer using the BD FACSDiva Software (BD Biosciences, San Jose, CA, EUA) and Flowjo Software 10 (Flowjo LCC, Ashland, OR, EUA). Ten thousand events were evaluated per experiment and cellular debris was omitted from the analysis.

#### Morphological analysis

To evaluate alterations in morphology, cells were cultured under coverslip and stained with May-Grunwald-Giemsa. Morphological changes were examined by light microscopy using Image-Pro software.

#### Annexin-V/PI staining assay

For apoptosis detection, we used the FITC Annexin V Apoptosis Detection Kit I (BD Biosciences), and the analysis was performed according to the manufacturer’s instructions. The cell fluorescence was determined by flow cytometry as described above. The protection assays using the caspase-3 inhibitor (Z-DEVD-FMK, BD Biosciences) and the antioxidant NAC (Sigma-Aldrich Co.) were performed. In brief, the cells were pre-treated for 2 h with 50 µM Z-DEVD-FMK and for 1 h with 5 mM NAC, followed by incubation with 4 µM of the complex for 48 h. The cells were then trypsinized and the FITC Annexin V Apoptosis Detection assay was conducted as described above.

#### Caspase-3 activation assay

A caspase-3 colorimetric assay kit (Sigma-Aldrich Co.) was used to investigate caspase-3 activation on complex-treated HCT116 cells based on the cleavage of DEVD-pNA and the analysis was performed according to the manufacturer’s instructions. Briefly, cells were lysed by incubation with cell lysis buffer on ice for 10 min and then centrifuged. Enzyme reactions were carried out in a 96-well flat-bottom microplate. To each reaction mixture, 5 μL cell lysate was added. Absorbance at 405 nm was measured using the SpectraMax 190 Microplate Reader (Molecular Devices). The results were expressed as specific activity (IU/mg protein) of caspase-3.

#### Measurement of the mitochondrial transmembrane potential

Mitochondrial transmembrane potential was determined by the retention of the dye rhodamine 123^30^. Cells were incubated with rhodamine 123 (5 μg/mL, Sigma-Aldrich Co,) at room temperature for 15 min in the dark and washed with saline. The cells were then incubated again in saline at room temperature for 30 min in the dark and cell fluorescence was determined by flow cytometry, as described above.

#### Measurement of cellular reactive oxygen species levels

The levels of ROS were measured according to previously described^31^ using DCF-DA (Sigma-Aldrich Co.). In brief, cells were treated with the complex for 1 and 3 h. Then, the cells were collected, washed with saline and re-suspended in FACS tubes with saline containing 5 μM DCF-DA for 30 min. Finally, the cells were washed with saline and the cell fluorescence was determined by flow cytometry, as described above.

#### DNA intercalation assay

DNA intercalation was assessed by examining the ability of the complex to displace ethidium bromide from ctDNA (Sigma-Aldrich Co.)^32^. The DNA intercalation assay was conducted in 96-well plate (100 µL) and contained 15 µg/mL ctDNA, 1.5 µM ethidium bromide and 5, 10 and 20 µM of complex in saline solution. The vehicle (0.1% DMSO) used for diluting the compound tested was used as the negative control. Doxorubicin (10 µM) was used as the positive control. Fluorescence was measured using excitation and emission wavelengths of 320 nm and 600 nm, respectively using the spectraMax Microplate Reader (Molecular Devices).

### Statistical analysis

Data are presented as mean ± S.E.M. or IC_50_ values and their 95% confidence intervals obtained by nonlinear regression. Differences among experimental groups were compared using analysis of variance (ANOVA) followed by the Student–Newman–Keuls test (*p* < 0.05). All statistical analyses were performed using GraphPad Prism (Intuitive Software for Science, San Diego, CA, USA).

## Electronic supplementary material

Supplementary Material
