# Supplementary material for: A ruthenium-based 5-fluorouracil complex with enhanced cytotoxicity and apoptosis induction action in HCT116 cells
Source: Sci Rep. 2018 Jan 10;8:288. doi: 10.1038/s41598-017-18639-6 (PMC5762908; doi:10.1038/s41598-017-18639-6)
Supplement: Supplementary file 1 — Supplementary Material [file 41598_2017_18639_MOESM1_ESM.doc]

**Supplementary Material**

**A ruthenium-based 5-fluorouracil complex with enhanced cytotoxicity and apoptosis induction action in HCT116 cells**

Valdenizia Rodrigues Silva^a,#^, Rodrigo S. Corrêa^b,#^, Luciano de Souza Santos^a,#^, Milena Botelho Pereira Soares^a,c^, Alzir Azevedo Batista^d^, Daniel Pereira Bezerra^a^,*

^a^Gonçalo Moniz Institute, Oswaldo Cruz Foundation (IGM-FIOCRUZ/BA), Salvador, Bahia, 40296-710, Brazil.

^b^Department of Chemistry, Federal University of Ouro Preto, Ouro Preto, Minas Gerais, 35400-000, Brazil.

^c^Center of Biotechnology and Cell therapy, Hospital São Rafael, Salvador, Bahia, 41253-190, Brazil.

^d^Department of Chemistry, Federal University of São Carlos, São Carlos, São Paulo, 13561-901, Brazil.

^#^ These authors contributed equally to this work

*** Corresponding author**

Prof. Dr. Daniel P. Bezerra, Gonçalo Moniz Institute, Oswaldo Cruz Foundation (IGM-FIOCRUZ/BA), Rua Waldemar Falcão, 121, Candeal, 40296-710, Salvador, Bahia, Brazil. E-mail: danielpbezerra@gmail.com Tel/Fax + 55 71 3176 2272

**Figure 1.** IR spectrum of the complex [Ru(5-FU)(PPh_3_)_2_(bipy)]PF_6_.

**Figure 2.** Cyclic voltammogram of the complex [Ru(5-FU)(PPh_3_)_2_(bipy)]PF_6_.


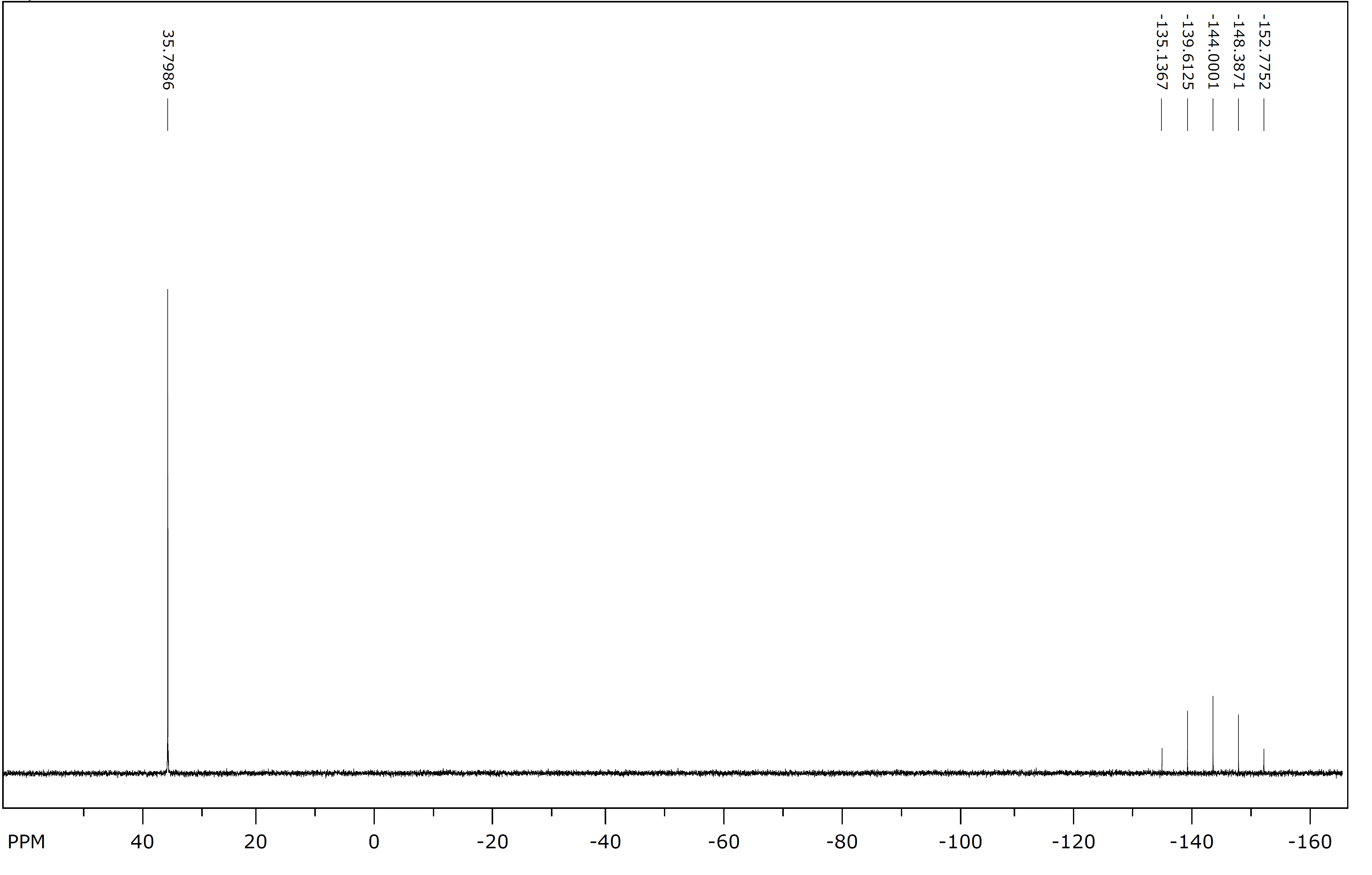


**Figure 3.** ^31^P{^1^H} MNR spectrum of the complex [Ru(5-FU)(PPh_3_)_2_(bipy)]PF_6_.


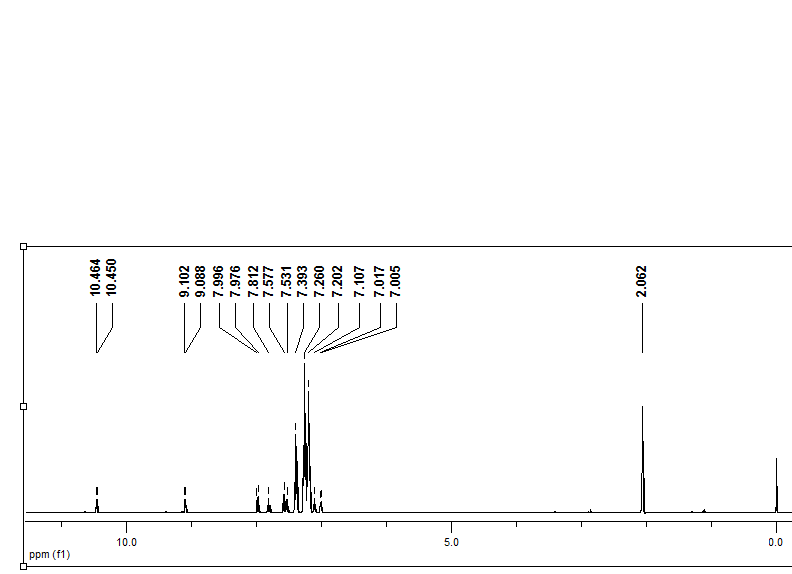


**Figure 4.** ^1^H MNR spectrum of the complex [Ru(5-FU)(PPh_3_)_2_(bipy)]PF_6_.


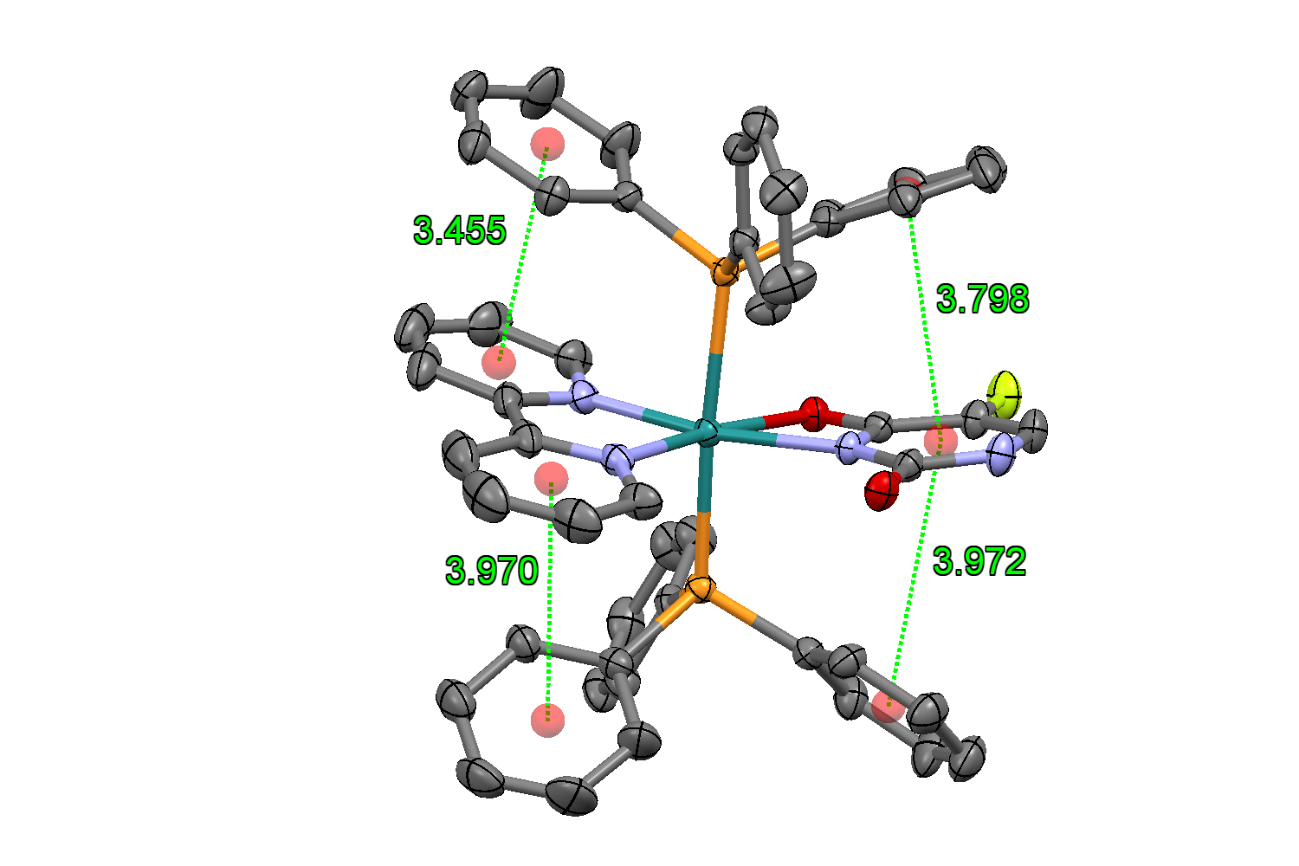


**Figure 5.** π-π contacts stabilizing the molecular structure of the complex [Ru(5-FU)(PPh_3_)_2_(bipy)]PF_6_.


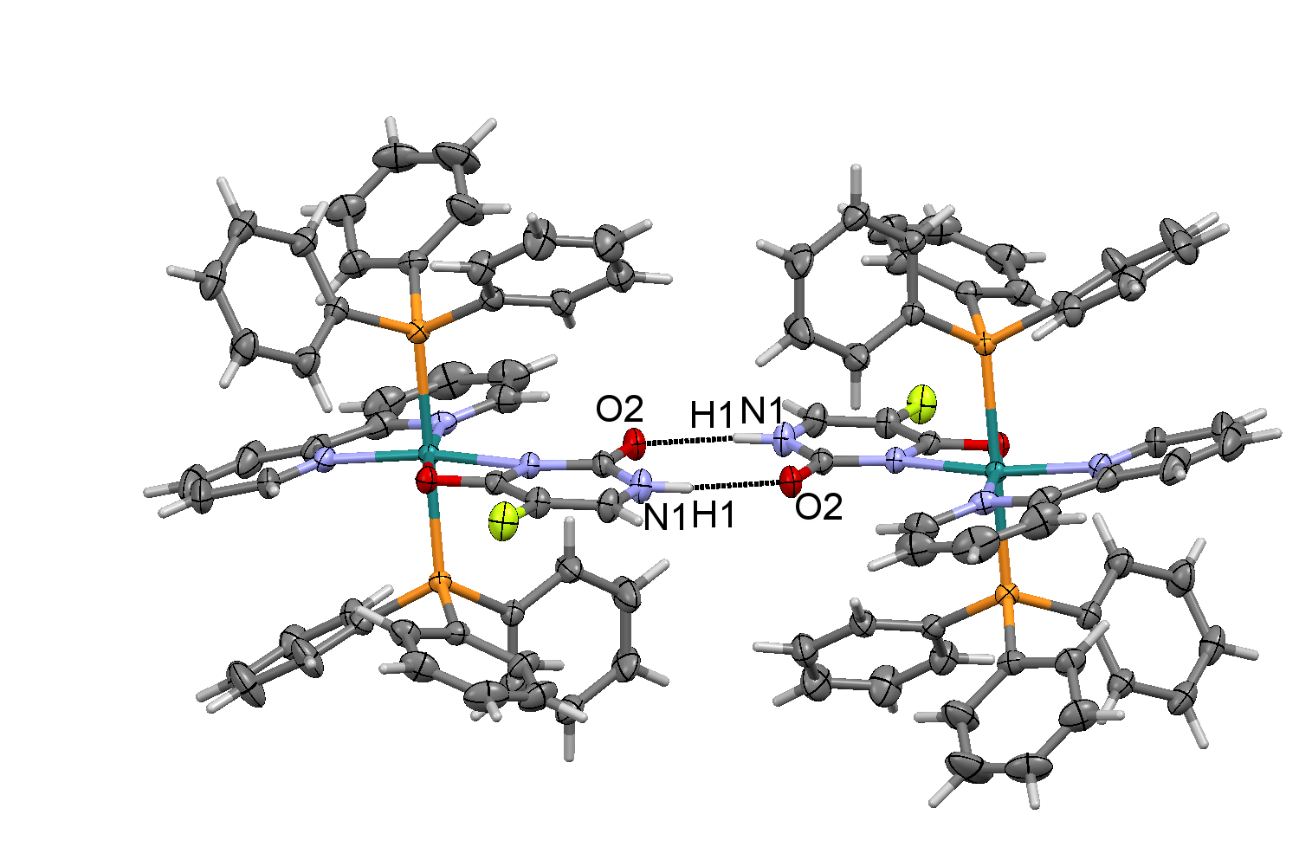


**Figure 6.** H-bonds forming centrosymmetric dimmers.

**Figure 7**. High resolution Mass spectrum of the complex [Ru(5-FU)(PPh_3_)_2_(bipy)]PF_6_.

**Table 1.** Infrared assignments for the complex [Ru(5-FU)(PPh_3_)_2_(bipy)]PF_6_.

| **Fragment** | **Band** |
| --- | --- |
| νNH | 3173 |
| νCH | 3078; 3055 |
| νCH | 3022, 2960 |
| ν_s_CH_3_ | - |
| ν­C=O | 1659 |
| ν­C=N | 1606 |
| ν­_as_­C=N_(ring)_ | 1600 |
| ν­C=C_(ring)_ | 1537 |
| ν­C=C_(ring)_ + νC=C_(PPh3)_ | 1481 |
| νC=N | 1435 |
| δ_s_­CH_3_ | - |
| δ_a_­CH_3_ | - |
| νC=C | 1329 |
| ν(C-N) | 1300;1270 |
| ν(C-F) | 1236 |
| *β*(C-H) | 1188, 1161 |
| ν_ass_P-CH/ νClO_4_ | 1092 |
| ν_ring_ | 1028 |
| ν_ring_ | 999 |
| ν P-F | 845 |
| γCH(φ) | 766 |
| γCH(φ) | 746 |
| γ anel | 698 |
| δP-CH | 617 |
| δ P-F | 557 |
| νRu-P | 519 |
| νRu-O | 496 |
| νRu-N | 464; 403 |

**Table 2.** Crystal data and structure refinement for the complex [Ru(5-FU)(PPh_3_)_2_(bipy)]PF_6_.

| Empirical formula | [C_50_H_40_F_7_N_4_O_2_P_3_Ru] |  |
| --- | --- | --- |
| Formula weight | 1055.84 |  |
| Temperature | 293(2) K |  |
| Wavelength | 0.71073 Å |  |
| Crystal system | Monoclinic |  |
| Space group | P2_1_/n |  |
| Unit cell dimensions | a = 13.0790(2) Å | α = 90°. |
|  | b = 18.2090(3) Å | β = 104.1640(10)°. |
|  | c = 20.5020(4) Å | γ = 90°. |
| Volume | 4734.23(14) Å^3^ |  |
| Z | 4 |  |
| Density (calculated) | 1.481 Mg/m^3^ |  |
| Absorption coefficient | 0.505 mm^-1^ |  |
| F(000) | 2144 |  |
| Crystal size | "0.35 x 0.19 x 0.18" mm^3^ |  |
| Theta range for data collection | 3.03 to 26.73°. |  |
| Index ranges | -16≤h≤16, -23≤k≤22, -25≤l≤25 |  |
| Reflections collected | 34484 |  |
| Independent reflections | 9935 [R(int) = 0.0372] |  |
| Completeness to theta = 26.73° | 98.9 % |  |
| Refinement method | Full-matrix least-squares on F^2^ |  |
| Data / restraints / parameters | 9935 / 0 / 617 |  |
| Goodness-of-fit on F^2^ | 1.014 |  |
| Final R indices [I>2sigma(I)] | R1 = 0.0457, wR2 = 0.1218 |  |
| R indices (all data) | R1 = 0.0568, wR2 = 0.1292 |  |
| Largest diff. peak and hole | 0.647 and -0.804 e.Å^-3^ |  |

**Table 3.** Selected bond lengths [Å] and angles [°] for the complex [Ru(5-FU)(PPh_3_)_2_(bipy)]PF_6_.

| Ru(1)-N(4) | 2.043(2) | N(2)-Ru(1)-P(1) | 93.78(7) |
| --- | --- | --- | --- |
| Ru(1)-N(2) | 2.049(2) | N(3)-Ru(1)-P(1) | 91.33(6) |
| Ru(1)-N(3) | 2.142(2) | O(4)-Ru(1)-P(1) | 85.22(5) |
| Ru(1)-O(4) | 2.1798(18) | N(4)-Ru(1)-P(2) | 93.55(7) |
| Ru(1)-P(1) | 2.3814(7) | N(2)-Ru(1)-P(2) | 87.88(7) |
| Ru(1)-P(2) | 2.4169(7) | N(3)-Ru(1)-P(2) | 86.08(6) |
| Ru(1)-C(4) | 2.568(3) | O(4)-Ru(1)-P(2) | 89.54(5) |
| O(4)-C(4) | 1.272(3) | P(1)-Ru(1)-P(2) | 174.76(2) |
| N(3)-C(4) | 1.350(4) | N(4)-Ru(1)-C(4) | 144.77(10) |
| N(3)-C(2) | 1.357(4) | N(2)-Ru(1)-C(4) | 136.93(10) |
| O(2)-C(2) | 1.243(4) | N(3)-Ru(1)-C(4) | 31.70(8) |
| F(1)-C(5) | 1.347(3) | O(4)-Ru(1)-C(4) | 29.67(8) |
| C(4)-C(5) | 1.426(4) | P(1)-Ru(1)-C(4) | 89.02(6) |
| N(1)-C(6) | 1.352(4) | P(2)-Ru(1)-C(4) | 86.38(6) |
| N(1)-C(2) | 1.373(4) | O(4)-C(4)-N(3) | 114.4(2) |
| C(6)-C(5) | 1.330(4) | O(4)-C(4)-C(5) | 126.5(3) |
| C(12)-C(13) | 1.397(5) | N(3)-C(4)-C(5) | 119.1(2) |
| C(12)-C(11) | 1.441(5) | C(6)-N(1)-C(2) | 123.1(3) |
| C(125)-C(124) | 1.367(6) | C(6)-N(1)-H(1) | 118.5 |
| C(11)-C(10) | 1.376(5) | C(2)-N(1)-H(1) | 118.5 |
| C(216)-C(215) | 1.398(5) | O(2)-C(2)-N(3) | 122.7(3) |
| C(225)-C(224) | 1.369(6) | O(2)-C(2)-N(1) | 121.0(3) |
| C(225)-C(226) | 1.383(5) | N(3)-C(2)-N(1) | 116.3(3) |
| C(136)-C(135) | 1.378(6) |  |  |
| N(4)-Ru(1)-N(2) | 78.17(11) |  |  |
| N(4)-Ru(1)-N(3) | 113.09(10) |  |  |
| N(2)-Ru(1)-N(3) | 167.51(10) |  |  |
| N(4)-Ru(1)-O(4) | 173.45(8) |  |  |
| N(2)-Ru(1)-O(4) | 107.74(9) |  |  |
| N(3)-Ru(1)-O(4) | 61.33(8) |  |  |
| N(4)-Ru(1)-P(1) | 91.66(7) |  |  |
